# Non-healing tongue ulcer in a rheumatoid arthritis patient medicated with leflunomide. An adverse drug event?

**DOI:** 10.4317/jced.53428

**Published:** 2017-02-01

**Authors:** Eleni-Marina Kalogirou, Nikolaos Katsoulas, Konstantinos I. Tosios, Andreas C. Lazaris, Alexandra Sklavounou

**Affiliations:** 1 DDS, MSc, Department of Oral Medicine and Pathology, Faculty of Dentistry, National and Kapodistrian University of Athens, Athens, Greece; 2 DDS, PhD, Assistant Professor, Department of Oral Medicine and Pathology, Faculty of Dentistry, National and Kapodistrian University of Athens, Athens, Greece; 3 MD, PhD, Professor, 1st Department of Pathology, Faculty of Medicine, National and Kapodistrian University of Athens, Athens, Greece; 4 DDS, MSc, PhD, Professor, Department of Oral Medicine and Pathology, Faculty of Dentistry, National and Kapodistrian University of Athens, Athens, Greece

## Abstract

Leflunomide is a member of the disease modifying anti-rheumatic drugs group used as a treatment modality in active rheumatoid and psoriatic arthritis. “Oral ulcers” are reported in 3-5% of leflunomide medicated rheumatoid arthritis patients with adverse events, but they are not described in detail in the literature. We present a case of an ulcer in the tongue of a rheumatoid arthritis patient managed with leflunomide and contemplate on its pathogenesis.

** Key words:**Leflunomide, oral ulcer, DHODH.

## Introduction

Leflunomide is a member of the disease modifying anti-rheumatic drugs group (DMARDs) and is approved by Food and Drug Administration as a treatment modality in active rheumatoid and psoriatic arthritis. Biochemically, it is a pro-drug that rapidly shifts to its active metabolite, teriflunomide or A77-1726 (1). Various molecular mechanisms of action for teriflunomide are described with inhibition of the mitochondrial enzyme dihydroorotate dehydrogenase (DHODH) anticipated when the drug is administered in immunomodulatory doses. DHODH is the key-enzyme in the de novo pyrimidine synthesis pathway, leading to ribo-nucleotide uridine monophosphate (rUMP) formation and promotion of cell proliferation through DNA and RNA synthesis enhancement. Therefore, teriflunomide inhibits proliferation of autoimmune T-cells and production of autoantibodies by B-cells (2).

Cutaneous ulcers are reported in rheumatoid arthritis patients and thought to have a multifactorial etiology, involving rheumatoid vasculitis, increased susceptibility of the skin to trauma due to alterations caused by prolonged systemic steroid treatment, and adverse effects of DMARDs and non-steroidal anti-inflammatory drugs (1-5). Leflunomide-associated cutaneous ulcers are considered rare, not preceded by trauma and show non-specific inflammation on microscopic examination (5,6). Adverse reactions of leflunomide in the oral cavity include oral candidiasis, herpetic infection (7) and “oral ulcers” (http://products.sanofi.us/arava/arava.html, revised February 2016), although the latter are not described in rheumatoid arthritis patients (8).

We present a case of an ulcer in the tongue of a rheumatoid arthritis patient medicated with leflunomide that microscopically showed severe epithelial dysplasia, and contemplate on its pathogenesis.

## Case Report

A 59-year-old woman was referred by her dentist for diagnosis and management of an ulcerated lesion on the tongue. According to the patient, the lesion was first noticed approximately 6 months before presentation and showed periods of “exacerbation”, lasting for less than 15 days, and “remission”. She considered it as a “common aphthous ulcer” and tried to manage it with local application of baking soda. Her medical history included rheumatoid arthritis with inflammatory polyarthritis diagnosed approximately 12 years ago and medicated with orally administered prednisolone 7.5 mg/day plus leflunomide (Arava®, Aventis Pharmaceuticals, Bridgewater, NJ) 20mg every day, for the last 6 years. She was, also, receiving olmesartan for hypertension and atorvastatin for hypecholisteraimia. She did not smoke or drink alcohol.

**Figure 1:**
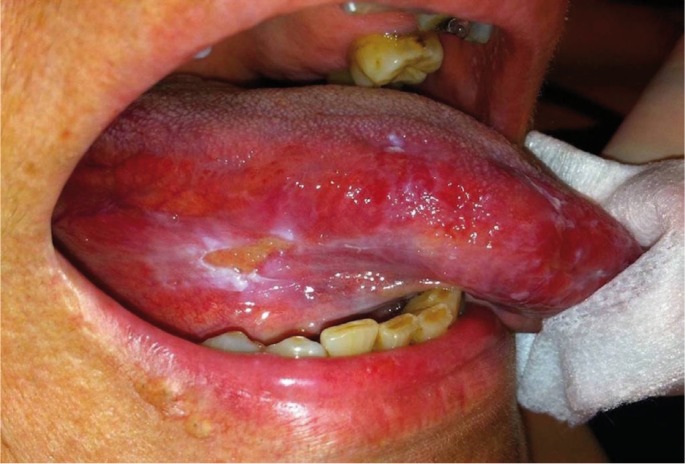
Irregularly shaped ulcer covered by fibrinopurulent membrane and partly surrounded by a firmly attached white plaque on the ventral surface of the right side of the tongue. Notice small, white epithelial shreds around the lesion.

Clinical examination revealed an irregularly shaped ulcer covered by fibrinopurulent membrane and partly surrounded by a firmly attached white plaque on the ventral surface of the right side of the tongue (Fig. 1). The ulcer measured approximately 1x0.5cm and was soft and slightly painful on palpation. Small, white epithelial tugs around the lesion were easily removed with gauze, while the rest of the oral mucosa was within normal limits. The lesion was in contact with broken amalgam fillings and decayed teeth’s roots. A provisional diagnosis of traumatic ulcer and/or lichenoid reaction was rendered and complete dental restoration was asked. In addition, oral rinses with a dexamethasone oral drops solution 2mg/ml x10ml and local applications of a chlorhexidine gel were prescribed for 2 weeks.

The patient presented for re-examination six weeks later. The amalgam fillings were replaced by composite resin fillings and the roots were extracted approximately 2 weeks ago, while she was still rinsing her mouth with the dexamethasone solution, but the lesions persisted. A partial biopsy was requested to establish a diagnosis, but she refused to proceed. At that time, on her rheumatologist’s advice he discontinued leflunomide and on re-examination a month later, a <50% healing of the ulcer was evident. Another month later, the ulcer had not fully healed and as reinstitution of leflunomide at a reduced dosing schedule (10mg/day) was necessary due to relapse of rheumatoid arthritis, she consented to the biopsy.

**Figure 2:**
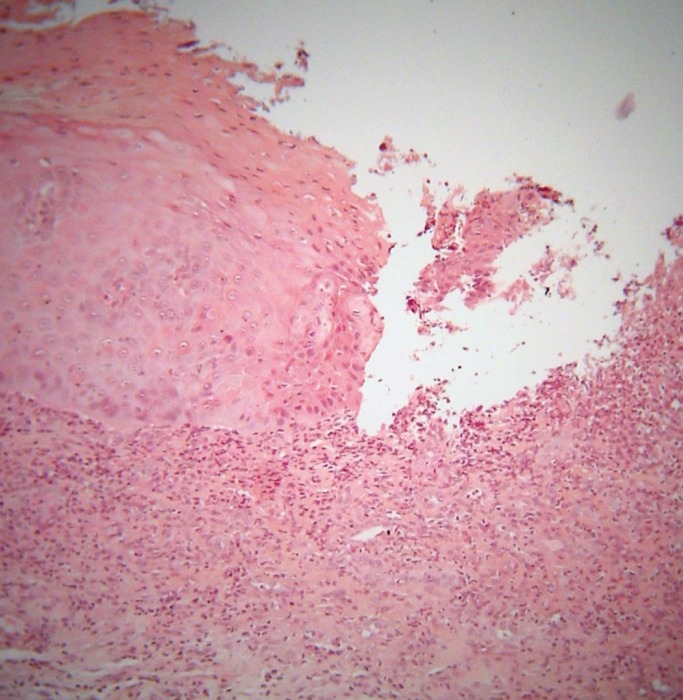
Ulceration covered by a fibrinopurulent membrane is surrounded by hyperparakeratotic and acanthotic stratified squamous epithelium [hematoxylin and eosin stain, original magnification x200].

**Figure 3:**
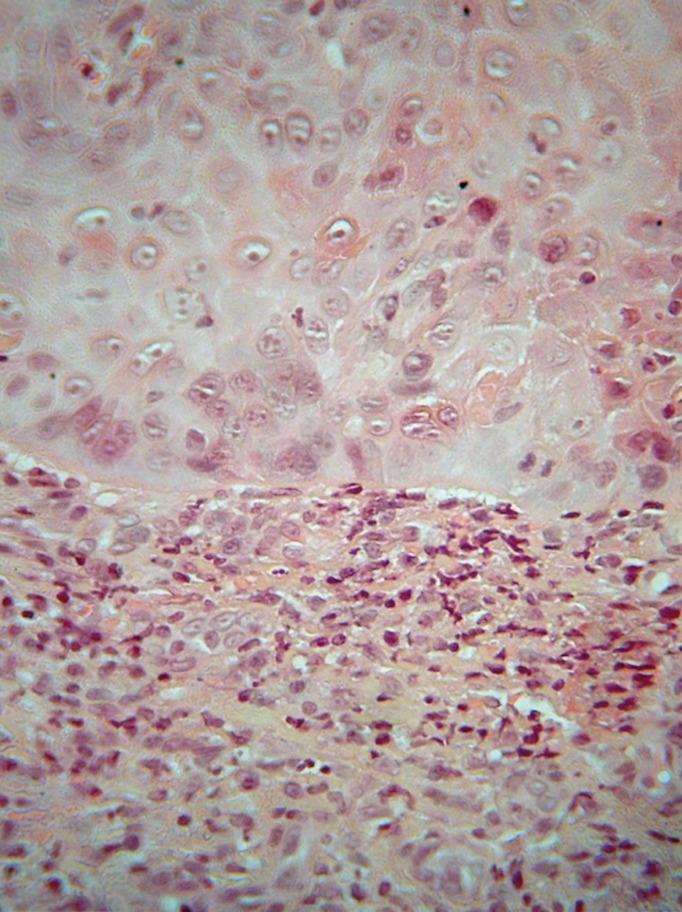
Atypical features of epithelial cells [hematoxylin and eosin stain, original stain, original magnification x400].

Microscopic examination of formalin-fixed and paraffin-embedded tissue sections showed an ulceration covered by a fibrinopurulent membrane and based by granulation tissue (Fig. 2). It was surrounded by hyperparakeratotic and acanthotic stratified squamous epithelium, showing drop-shaped rete ridges, irregular stratification, loss of polarity of basal cells, increased nuclear to cytoplasmic ratio, cellular pleomorphism, increased nuclear size, increased number and size of nucleoli, dyskeratosis, as well as increased number of mitotic figures (Fig. 3). Those features occupied the full extent and thickness of the epithelium. The underlying connective tissue showed dense inflammatory infiltrates of lymphocytes, plasma cells and polymorphonuclears, mostly in a band-like subepithelial distribution. Atypical cell were not found in the connective tissue. A periodic acid-Schiff (PAS) stain did not reveal candidal hyphae. A histopathologic diagnosis of severe epithelial dysplasia was rendered. Therefore, excision of the residual white lesions on clinically healthy margins was performed, confirming the initial diagnosis. The healing process was uneventful, and six months later the patient is on 20mg/day of leflunomide, while no recurrence or new lesions have developed. The patient gave her informed consent for the use of her data for study.

## Discussion

“Oral ulcers” are described in 3-5% of leflunomide-medicated rheumatoid arthritis patients reporting adverse events (http://products.sanofi.us/arava/arava.html, revised February 2016), but this may be a sign of various oral diseases, including oral candidiasis and herpetic infection that may be seen in those patients (7).

In the case described herein, the presence of amalgam fillings in close proximity to the lesion raised the possibility of a lichenoid amalgam reaction, but after amalgam replacement by composite resin no healing of the lesion was evident, while the histologic features of the epithelium were not consistent with this diagnosis (9). Leflunomide use in rheumatoid arthritis may be rarely associated with infections, like oral candidiasis and herpetic infection (7), but neither the clinical presentation nor the microscopic features were consistent with a fungal or herpetic disease (9). The ulcer was clearly associated with injurious factors, was su-rrounded by hyperkeratotic mucosa as seen in chronic traumatic ulcers, while epithelial tugs of traumatic etiology were seen in its vicinity. Therefore, it was initially considered consistent with a chronic traumatic ulcer, but it did not heal following removal of all possible injurious factors (9). Withdrawal of leflunomide for a month resulted in <50% healing, therefore we consider that the ulcer was induced by the local trauma and that healing was inhibited by leflunomide.

In particular, the rapid proliferation that is necessary for re-epithelialization of a traumatic ulcer requires a significant increase in pyrimidine ribonucleotides levels, mostly rUMP, in order for the cells to synthesize DNA and progress from G1 through the S phase of the cell cycle (2). DHODH inhibition hampers pyrimide synthesis, causes G1 phase arrest and restrains healing, although in therapeutic doses for rheumatoid arthritis it does not cause oral mucositis, as proliferating cells may use salvage pathways for pyrimidine synthesis (3). In addition, teriflunomide has the ability to inhibit epidermal growth factor-depended cell growth (10).

This pathogenetic mechanism may also explain the presence of epithelial dysplasia around the ulcer, although the diagnosis of an ulcerated oral leukoplakia cannot be fully excluded. Alterations similar to oral epithelial dysplasia are seen in oral ulcers after chronic methotrexate administration or folate deficiency and described as “pseudo-dysplasia” (11). They are caused by the long-standing effect of drug or non-drug-induced folate deficiency on DNA synthesis and cellular maturation mechanisms, as methotrexate mainly inhibits the enzyme dihydrofolate reductase and other enzymes in the folate pathway, leading to purine and pyrimidine synthesis disruption and inhibition of cell cycle (11). Interestingly, rUMP and other ribonucleotides inhibited by leflunomide are in part responsible for synthesis of deoxyribonucleotides (2). As there are no yet diagnostic features for differentiating dysplasia from “pseudo-dysplasia” this hypothesis requires further investigation.

Oral ulcers in rheumatoid arthritis patients medicated with leflunomide may be attributed to disturbed re-epithelialization due to the action of the drug on epithelial cell proliferation. The detailed description of more cases of “oral ulcers” in those patients is necessary for documenting that oral ulceration is an adverse event associated with immunomodulatory doses of leflunomide.
